# Eosinophilic granulomatosis with polyangiitis: myocardial thickening reversed by corticosteroids

**DOI:** 10.1186/s12872-017-0734-8

**Published:** 2017-12-20

**Authors:** Gustav Mattsson, Peter Magnusson

**Affiliations:** 1 Centre for Research and Development, Uppsala University/Region Gävleborg, SE-801 87 Gävle, Sweden; 2 Cardiology Research Unit, Department of Medicine, Karolinska Institutet, SE-171 76 Stockholm, Sweden

**Keywords:** Cardiac imaging, Cardiac magnetic resonance, Cardiomyopathy, Churg-Strauss, Corticosteroids, Echocardiography, Eosinophilic granulomatosis with polyangiitis, Heart failure, Hypertrophy, Myocardial thickening

## Abstract

**Background:**

In 1951 Churg and Strauss first described the clinical condition now known as eosinophilic granulomatosis with polyangiitis (EGPA), characterized by asthma, nasal polyposis, rhinosinusitis, hypereosinophilia with organ infiltration, and necrotizing vasculitis. It is classified as an antineutrophil cytoplasmic antibody (ANCA) associated vasculitis, but ANCA negativity is common and more frequently encountered in EGPA with myocardial involvement. Long-term survival has substantially improved with corticosteroid treatment but myocardial involvement is still the leading cause of death in EGPA.

**Case presentation:**

A 53-year old man with a history of asthma and nasal polyposis presented with acute chest pain and elevated troponin; a percutaneous coronary intervention was performed. The left ventricle was described as *hypertrophic*. After 20 days the myocardium had markedly increased in thickness of both the right and left ventricle. Evaluation revealed hypereosinophilia in the blood and nasal mucosal tissue, which confirmed the diagnosis of EGPA. He presented with signs of active vasculitis including weight loss, tiredness, intracerebral hemorrhage, and increasing serum creatinine. After 6 days of corticosteroid treatment, the myocardium returned to its initial thickness.

**Conclusion:**

Rapid and marked thickening of the myocardium is not frequently reported but may occur in EGPA. Myocardial thickening in EGPA can be quickly reversed by corticosteroids, and is most likely caused by edema.

## Background

Eosinophilic granulomatosis with polyangiitis (EGPA), formerly Churg-Strauss syndrome, is a necrotizing vasculitis affecting small to medium vessels [1, 2]. It is linked to antineutrophil cytoplasmic antibodies (ANCA) and is therefore classified as an ANCA-associated vasculitis together with granulomatosis with polyangiitis (formerly Wegener’s granulomatosis) and microscopic polyangiitis. Histologically, EGPA is characterized by extravascular granulomas and eosinophilic infiltrates; because hypereosinophilia is evident in the blood, EGPA has been classified as a hypereosinophilic syndrome [2, 3]. The typical clinical presentation of EGPA includes a prodromal phase that lasts for years and consists of asthma, nasal polyposis, and chronic pansinusitis. In the next phase, organ involvement due to eosinophilia is seen, typically in the lungs, heart, and gastrointestinal tract. Later, vasculitis may affect the peripheral nervous system, kidneys, and skin [3, 4].

The prevalence of EGPA has been reported to be 10.7 per 1,000,000 in adults [5]. Contrary to other vasculitides, EGPA does not appear to be more common in Caucasians [5]. Prognosis has improved greatly since the introduction of corticosteroids; five-year relapse-free survival has been estimated to be 58% and 68% for ANCA positive and negative patients, respectively [6]. In approximately 40% of EGPA cases ANCA is positive, mainly myeloperoxidase but also proteinase 3 ANCA [7].

## Case presentation

A 53-year-old man presented at the emergency department with chest pain, radiating towards his left shoulder, which had lasted for half an hour. ECG showed signs of left ventricular hypertrophy but similar abnormalities had been noted 2 years earlier in conjunction with surgery for nasal polyps. The patient had a history of asthma diagnosed 5 years ago, celiac disease, ulcerative colitis treated with sulfasalazine, and anemia treated with cyanocobalamin and folic acid. At arrival, troponin T was elevated at 0.469 ng/mL, but did not show any dynamic change. Medications for acute coronary syndrome were started and he underwent a percutaneous coronary intervention without stenting to address a suspected occlusion of the left posterior descending artery. Echocardiography revealed hypertrophy of the left ventricle, suspected mild hypertrophy of the right ventricle, and a normal ejection fraction. At discharge he was prescribed atorvastatin, bisoprolol, acetylsalicylic acid, and clopidogrel instead of ticagrelor due to dyspnea.

**Fig. 1:**
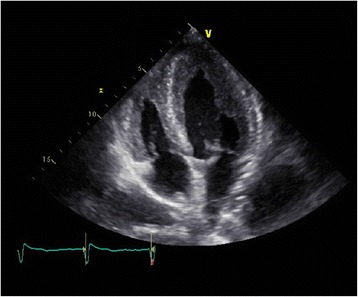
Transthoracic echocardiography, 2 days before initiation of corticosteroid treatment; apical four-chamber view showing biventricular thickening of the myocardium most prominent towards the apex

Chest pain recurred 20 days after discharge and while at home the patient had experienced tiredness, general weakness, dyspnea, loss of appetite, and weight loss. Troponin T was once again elevated but without dynamic changes and N-terminal pro-B-type natriuretic peptide increased to 13,377 pg/mL. A computed tomography of the thorax and abdomen was performed to rule out malignancy. Due to dysphagia and dysarthria, a computed tomography of the head demonstrated a minor intracerebral hemorrhage in the left parietal lobe and pansinusitis, following this antiplatelet therapy was discontinued. Echocardiography showed a marked increase in biventricular thickness compared to 3 weeks earlier, as well as a decreased biventricular ejection fraction (Fig. 1). Notably, his eosinophilic count was 3800/μL. It was decided to transfer the patient to a tertiary center for further evaluation.

EGPA was suspected so the patient started pulse treatment with high-dose intravenous methylprednisolone 250 mg three times daily and changed after 3 days to prednisolone 60 mg daily. The patient was ANCA negative. A new computed tomography showed a low attenuating area in the right frontal lobe of the brain, and angiography revealed a thin vertebral artery on the left side. A bone marrow biopsy showed marked eosinophilia, but no signs of primary bone marrow disease. In fact, prior colon biopsies had also shown eosinophilic infiltrates. Serum creatinine levels were increasing from previously normal values up to 132 μmol/L, which suggested renal involvement. A fat tissue biopsy ruled out amyloidosis.

**Fig. 2:**
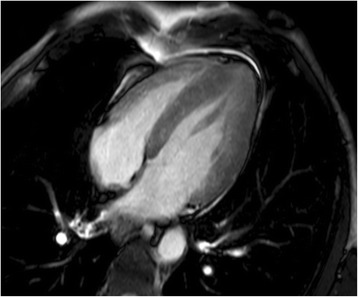
Cardiac magnetic resonance tomography, after 4 days of corticosteroid treatment; edema sensitive sequence, four-chamber view showing regression of myocardial thickening with no signs of edema

**Fig. 3:**
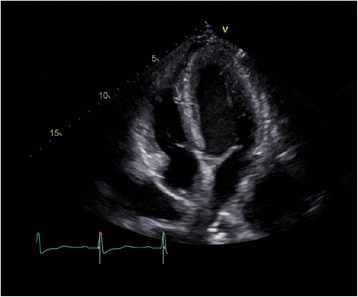
Transthoracic echocardiography, after 6 days of corticosteroid treatment; apical four-chamber view showing marked regression of the myocardial thickening previously seen

Biventricular hypertrophy was confirmed by cardiac magnetic resonance tomography (Fig. 2) performed after 4 days of corticosteroid treatment. Generalized fibrosis was present but no edema or regional hypokinesia. After 6 days of corticosteroid treatment, hypertrophy was in regression on echocardiography (Fig. 3). Endomyocardial biopsy after 6 days of corticosteroid therapy showed myocyte necrosis but no signs of active inflammation. A nasal mucosal biopsy demonstrated necrosis with inflammatory infiltrates of plasma cells and eosinophils. It was then decided to add cyclophosphamide to the treatment and the eosinophilic count had normalized. At discharge the patient reported remarkable improvement and claimed he felt better than he had had for a several years.

## Discussion and conclusions

The American College of Rheumatology established criteria for the diagnosis of EGPA in 1990, of which four out of six are sufficient for diagnosis: asthma, eosinophilia (>10%), neuropathy, pulmonary infiltrates, paranasal sinus abnormality, and extravascular eosinophils [8]. Our patient had a prodromal phase with adult-onset asthma, sinusitis, and repeated surgery for nasal polyposis. During evaluation, hypereosinophilia (33%) in the blood and eosinophilic infiltrates in the nasal mucosa were observed. He had been diagnosed with ulcerative colitis at the age of 18. Biopsies taken from his colon 2 years earlier already showed eosinophilic infiltrates, indicating that he had been in the eosinophilic phase for considerable time before presenting at the emergency department. This was supported by the fact that around the same time, signs of left ventricular hypertrophy were observed on the ECG, which suggested cardiac involvement of EGPA. Corticosteroid treatment was initiated before the endomyocardial biopsy was performed, which explains why necrosis but no sign of active inflammation was evident.

This patient did not meet two of the diagnostic criteria, namely peripheral neuropathy and lung infiltrates, but these are the least sensitive criteria [8]. Notably, he had a cerebral hemorrhage, likely due to the presence of ischemic lesions in combination with antiplatelet therapy. Vasculitis affecting the central nervous system and leading to hemorrhagic stroke is uncommon in EGPA, but has been reported [9–11]. One case report also described concomitant vertebral artery dissection [11]. The increase in serum creatinine lead to high suspicion of renal involvement and creatinine levels normalized after initiation of treatment. Since it was believed that the results of a kidney biopsy would not affect treatment or outcome, it was not performed owing to the associated risks, which are not negligible. Renal involvement in EGPA is present in approximately one quarter of cases, however it is less common in ANCA-negative patients [12].

The absence of ANCA is linked to eosinophilic organ involvement, while ANCA positivity predisposes to vasculitis [6, 13]. Even though ANCA negativity by itself is a favorable prognostic factor with lower risk of remission, cardiac involvement is more common than in ANCA-positive patients [13]. Cardiac involvement is the leading cause of mortality in EGPA [6, 14, 15]. The first echocardiogram in our patient showed moderate left ventricular hypertrophy in the absence of abnormal loading conditions, such as hypertension or aortic stenosis; furthermore, there were no other signs of hypertrophic cardiomyopathy. Upon presentation, the patient appeared to have suffered a general deterioration with weakness, tiredness, and weight loss; this together with myocardial ischemia, intracerebral hemorrhage, and increasing serum creatinine should be interpreted as the patient entering the vasculitis phase of EGPA. Signs of cardiac involvement described in the literature include: heart failure, reduced ejection fraction, pericardial effusion, ECG abnormalities, ventricular arrhythmia, pulmonary artery hypertension, intraventricular thrombi, elevated troponin, and mitral-, aortic-, and tricuspid valve insufficiency [3, 16]. Cardiac magnetic resonance tomography with late gadolinium enhancement can visualize fibrosis, typically subendocardial; endomyocardial biopsy can show fibrosis, necrosis, and eosinophilic infiltrates [16, 17]. In this case, echocardiography revealed a sudden and marked myocardial thickening that was quickly reversed by corticosteroid treatment. Thickening of the myocardium in EGPA and its reversal at 3 months follow-up after treatment with corticosteroids and cyclophosphamide has been previously described [18]. In our case, the acute onset of swelling most likely was explained by edema in addition to eosinophilic infiltration. In myocarditis ventricular wall thickening is sometimes seen, it is caused by interstitial edema with myocyte diameter remaining unchanged [19]. Endomyocardial biopsy was performed after 6 days of corticosteroid regimen which explains why no active inflammation was seen. Interestingly, cardiac magnetic resonance tomography after 4 days of corticosteroids showed no clear signs of edema. We interpret this as rapid regression of edema after treatment, whereas residual myocardial thickening was likely explained by eosinophilic infiltrates.

Owing to the patient’s rapid deterioration, corticosteroid treatment was commenced before biopsies could be performed. The EGPA Consensus Task Force recommendations were followed: intravenous methylprednisolone 7.5–15 mg/kg/day in the presence of life-threatening symptoms followed by prednisolone 1 mg/kg/day [15]. According to the Five Factor Score based on the French Vasculitis Study Group used to offer the prognosis for ANCA-associated vasculitides, each of the following count as one point: age > 65 years, cardiac involvement, gastrointestinal manifestations, creatinine >150 μmol/L, and the absence of ear/nose/throat manifestations [20]. In patients scoring ≥1 point, an immunosuppressant in addition to corticosteroids is recommended [15, 20]. Gastrointestinal involvement in the Five Factor Score involves severe manifestations, such as bleeding, perforation, or pancreatitis; eosinophilic infiltration does not count. Our patient scored 1 point due to cardiac involvement and because of this he was started on cyclophosphamide.

In conclusion, EGPA can present with rapid myocardial thickening, likely due to myocarditis and edema. This case report demonstrates that corticosteroids, the mainstay of EGPA therapy, can reverse this thickening in as few as 6 days. Early diagnosis is crucial, therefore evaluation in patients with suspected EGPA includes echocardiography, cardiac magnetic resonance, endomyocardial biopsy in addition to careful history taking and laboratory assessment.

## Abbreviations

ANCA: Antineutrophil cytoplasmic antibody
EGPA: Eosinophilic granulomatosis with polyangiitis
